# Tree Spatial Structure, Host Composition and Resource Availability Influence Mirid Density or Black Pod Prevalence in Cacao Agroforests in Cameroon

**DOI:** 10.1371/journal.pone.0109405

**Published:** 2014-10-14

**Authors:** Cynthia Gidoin, Régis Babin, Leïla Bagny Beilhe, Christian Cilas, Gerben Martijn ten Hoopen, Marie Ange Ngo Bieng

**Affiliations:** 1 Supagro, UMR System, Montpellier, France; 2 CIRAD, UPR Bioagresseurs, Montpellier, France; 3 icipe, Plant Health Division, Coffee Pest Project, Nairobi, Kenya; 4 IRAD, Yaoundé, Cameroon; 5 CIRAD, UMR System, Montpellier, France; Bangor University, United Kingdom

## Abstract

Combining crop plants with other plant species in agro-ecosystems is one way to enhance ecological pest and disease regulation mechanisms. Resource availability and microclimatic variation mechanisms affect processes related to pest and pathogen life cycles. These mechanisms are supported both by empirical research and by epidemiological models, yet their relative importance in a real complex agro-ecosystem is still not known. Our aim was thus to assess the independent effects and the relative importance of different variables related to resource availability and microclimatic variation that explain pest and disease occurrence at the plot scale in real complex agro-ecosystems. The study was conducted in cacao (*Theobroma cacao*) agroforests in Cameroon, where cocoa production is mainly impacted by the mirid bug, *Sahlbergella singularis*, and black pod disease, caused by *Phytophthora megakarya.* Vegetation composition and spatial structure, resource availability and pest and disease occurrence were characterized in 20 real agroforest plots. Hierarchical partitioning was used to identify the causal variables that explain mirid density and black pod prevalence. The results of this study show that cacao agroforests can be differentiated on the basis of vegetation composition and spatial structure. This original approach revealed that mirid density decreased when a minimum number of randomly distributed forest trees were present compared with the aggregated distribution of forest trees, or when forest tree density was low. Moreover, a decrease in mirid density was also related to decreased availability of sensitive tissue, independently of the effect of forest tree structure. Contrary to expectations, black pod prevalence decreased with increasing cacao tree abundance. By revealing the effects of vegetation composition and spatial structure on mirids and black pod, this study opens new perspectives for the joint agro-ecological management of cacao pests and diseases at the plot scale, through the optimization of the spatial structure and composition of the vegetation.

## Introduction

Maintaining production and improving ecosystem services of agro-ecosystems while simultaneously reducing dependence on external inputs such as pesticides and fertilizers is a major challenge in agriculture today [1]. To achieve this goal, two concepts based on the “intensification in the use of the natural functionalities that ecosystems offer” are ecological intensification and agro-ecology [2]. A central idea behind these concepts is that associated biodiversity could provide a large range of ecosystem services that may have the same effect as chemical inputs, including pesticides, for pest regulation [3]. With regard to pest and disease regulation services, combining crop plants with other plant species can impact pest and disease occurrence through divers and often complex mechanisms [4]–[5]. However, the regulation of pests and diseases depends much more on specific characteristics of plant associations than on species richness *per se*, yet these characteristics are largely unknown.

Variability in resource availability is one of the main hypotheses proposed to explain the impact of plant diversity on pest and disease occurrence in agro-ecosystems [5]–[6]. Two mechanisms are involved in this hypothesis: (i) the “resource dilution” mechanism, which consists of a reduction in pest and disease occurrence through a decrease in host abundance [7] and (ii), the “alternate resource introduction” mechanism, which consists of an increase in pest and disease occurrence through the introduction of alternative hosts [8]. In general, “resource dilution” holds true for specialist pests and diseases that have only one or a few host plants [9], while the “alternate resource” mechanism generally holds true for more generalist pests and diseases.

Another mechanism involved in pest and disease regulation in multispecies agro-ecosystems is linked to variations in microclimatic conditions [6]. For example, shade trees associated with a crop reduce light availability and wind speed, buffer temperature, and increase relative humidity [10]. These microclimatic variations, which can be grouped under the term shading [11], are determined by several factors, including the spatial structure of shade trees. Tree spatial structure can be described by the characteristics of its vertical and horizontal structure including the number of strata, the density of individual trees in a stratum, and the horizontal distribution of shade trees [4]. These characteristics determine the mean and variance of sunlight transmitted to the understory and can directly affect processes related to the life cycles of pests and pathogens of the crop [6]–[8]–[12].

Under natural conditions, the microclimatic and resource alteration mechanisms are interlinked and interact. For example, microclimatic variations affect the vegetative growth of host plants and hence the availability of sensitive tissue resources for pests and diseases [13]–[14]. However, microclimatic and resource alteration mechanisms are rarely studied together and, as a result, their independent effects and relative importance in pest and disease occurrence at the plot scale are still not known. Although these mechanisms have been confirmed by both empirical research on plant pests and diseases [15] and by epidemiological models [16], the present study is one of the first to try to assess the relative importance of these different mechanisms in a real complex agro-ecosystem. Furthermore, depending on their biology, pests and diseases are differently impacted by each of these mechanisms [17]. In a previous work in cacao agroforest plots in Costa Rica, we studied the independent and joint effects of variables related to the two mechanisms on the impact of one cacao fungal disease (frosty pod rot caused by *Moniliophthora roreri*) [18]. The results of this study revealed the significant influence of the spatial structure of shade trees in the regulation of cacao disease.

In this paper, we focus on cacao (*Theobroma cacao* L.) agroforests in Cameroon. An agroforest is defined as an agro-ecosystem that is structurally close to a natural forest ecosystem with diverse vegetation composition and complex spatial structure [19]. Cameroon is currently the fifth cocoa producer of the world [20], but cocoa production is seriously affected by the mirid bug *Sahlbergella singularis* Hagl. [21] and black pod disease, caused by *Phytophthora megakarya* Brasier & Griffin [22]. Cocoa mirids are not specific to the crop and can be found on several plant species belonging to the Malvaceae family (e.g. *Ceiba pentandra*, *Cola acuminata*, *Cola lateritia* and *Sterculia rhinopetala*) [23], some of which are often associated with cacao trees in agroforests [21]. Black pod disease (BP) caused by *Phytophthora megakarya* can be considered as a specialized pathogen of cacao trees since it does not damage other plant species present in cacao based agroforests and has not been isolated from diseased plant material outside cacao based agroforests [24]–[25]. *Phytophthora megakarya* zoospores are dispersed by rain splash between 5–6 meters [26] and may spread further when free flowing water is available. Adult mirids are able to fly 1–2 km on average [27] but the larvae cannot fly.

The balance of shading effects on pest and disease regulation can be complex [4]–[8], as is the case in the regulation of mirids and black pod disease. Firstly, heavy shade reduces mirid density, probably by limiting cacao vegetative growth, i.e. the availability of young shoots (also named flushes), which are a primary food resource for mirids [21]. At the same time, the introduction of shade trees belonging to the Malvaceae family, known to include alternative host plants for mirids, could reduce the positive impact of shading on mirid density [28]. In the case of black pod, shading is considered to provide microclimatic conditions favorable to the spread and development of the disease [29]. However, the introduction of shade trees may result in resource dilution through a decrease in cacao tree abundance and hence in the availability of potentially sensitive tissue (mainly cacao fruits, also known as cacao pods), which could then reduce the negative impact of shading on the regulation of black pod. Therefore, assessing the respective effects of host composition, the availability of sensitive tissue, and shade tree spatial structure is particularly important as it could improve the joint integrated management of mirids and black pod disease in cacao agroforests.

The question we addressed in this study was “Which part of the variation in mirid density and black pod prevalence among agroforests is explained by host composition, the spatial structure of the vegetation, and resource availability?” To answer this question, mirid density, black pod prevalence and key variables related to spatial structure and composition of the vegetation and the amount of available sensitive tissue were characterized in 20 cacao agroforest plots in the region Centre in Cameroon. We examined the independent contributions of key variables explaining mirid density and disease prevalence using a hierarchical partitioning protocol. We then discuss the implications of our results for the joint agro-ecological management of mirids and black pod disease in cacao agroforests through the optimization of the spatial structure and composition of the vegetation.

## Materials and Methods

### Study site and sampling plots

**Figure 1:**
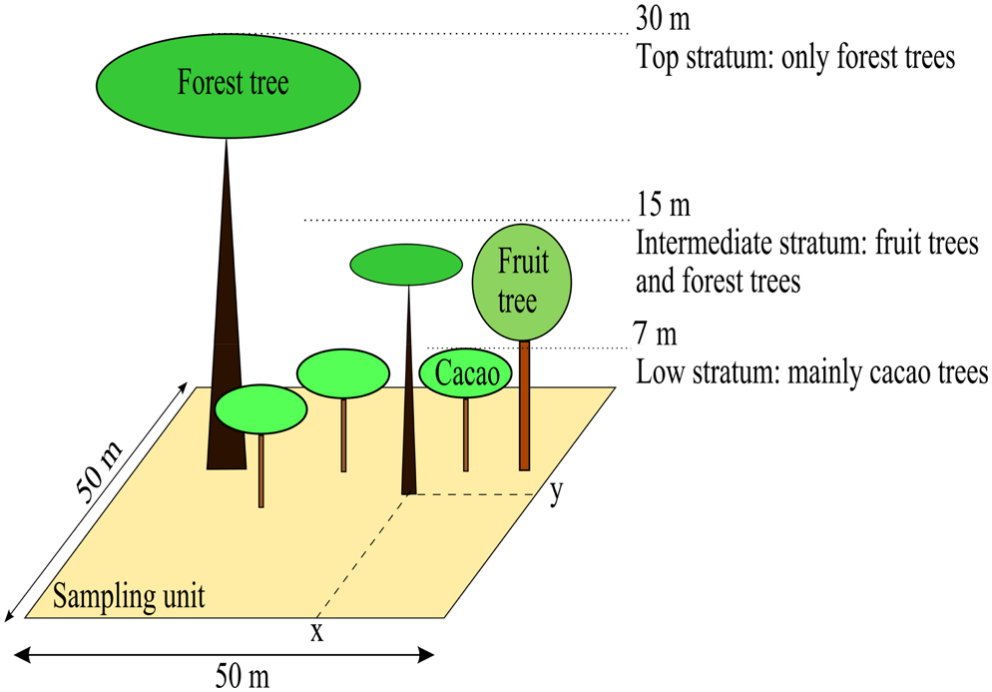
Schematic representation of the vertical structure of cacao agroforest in the Lékié department in Cameroon.

The Lékié department in Cameroon was chosen because it represents an intermediate situation on the north–south gradient characterizing natural conditions in the cocoa growing region of central Cameroon [30]. In this department, cacao agroforests are located in a mosaic of cacao based agroforestry plantations owned by different farmers and of forest zones with substantial human activity. Individual farmers generally own one or two cacao plantations with an average size of 2.69 ha (SD = 0.09, n = 687) [30]. Twenty 2,500 m^2^ sampling units (50×50 m, see Figure 1) were selected in 20 cacao agroforest plantations belonging to different farmers’, based on the criterion that the unit contained more than 10 shade trees associated with the cacao trees. Each sampling unit (hereafter referred to as ‘plot’) was representative of the cacao agroforest plantation as a whole. The 20 plots were located in a total area of 10×10 km spread over three localities: Nkolobang (9 plots), Zima (7 plots) and Mbakomo (4 plots). The rainfall pattern is bimodal with between 1,500 and 1,600 mm of rainfall per year [30]. Data were collected between September 2011 and November 2012.

### Characterization of vegetation structure

In September 2011, vegetation composition and spatial structure were characterized in each plot. The x,y cartesian coordinates of each plant (2 m tall or over) were recorded in each plot using a theodolite (Leica Builder 409, Leica Geosystems, Heerbrugg, Switzerland). Plants were identified to either species or family level, and in a few cases, were not identified. The trees associated with the cacao trees, whether identified or not, were classified in two categories: forest trees or fruit trees. This classification is based on the management of trees in agroforests and is that used in the literature, it states that the fruit trees are mainly introduced by farmers whereas the forest trees are mainly left standing in agroforests when the cacao plantation is established [19]–[31].

Pest and disease resources were described using variables related to host composition. The cacao tree was the main host for mirids and black pod in the agroforests we studied. For that reason, the relative abundance of cacao trees (*Abca*, i.e. their abundance with respect to all the vegetation mapped in the plot) was assumed to be a valid proxy to study the resource dilution mechanism. For mirids, alternative hosts to cacao trees were present in the agroforests concerned [23] so the presence/absence of alternative mirid hosts (*Alter*) was also taken into account. Concerning black pod, in our study, the cacao tree was considered to be the only host.

Microclimatic conditions were described using variables related to spatial structure of the vegetation. The vertical and horizontal structure of the cacao trees and associated shade trees (forest trees and fruit trees) affect mean and variance of the light transmitted to the pest and disease present in the understory. The vertical spatial structure was characterized using four strata: the top stratum (at a distance of approximately from 15 to 30 m from the ground); the intermediate stratum (approximately 7 to 15 m from the ground), the low stratum with vegetation in the same stratum as cacao trees (approximately 3 to 7 m from the ground) and the lowest stratum with vegetation lower than cacao trees (approximately 2 to 3 m from the ground) (Figure 1). This classification is justified by field observations to distinguish the amount of shade received by the cacao trees (the top and intermediate canopy strata provided shade for the cacao trees) and the self-shading produced by cacao trees and the vegetation in the same strata. Each plant was recorded as belonging to one of these strata. The total cover (*Dtot*) produced by the cacao trees (self-shading) and associated shade trees was estimated by the total tree density from the cacao to the top canopy strata (the lowest stratum was not included in this calculation). The shade tree cover (*Dsha*) was estimated by the sum of tree densities in the top and intermediate canopy levels. The proportion of shade tree cover provided by the intermediate stratum was estimated by the proportion of shade trees in the intermediate stratum compared with the total number of shade trees (%*Inter*).

The horizontal spatial structure, which is rarely taken into account in agro-ecological studies, was characterized using the *L*(r) function (Besag in Ripley [32]). Although this function is often used in forest research [14] it has rarely been used for agro-ecosystems. The *L*(r) function is based on the calculation of the expected number of neighbors within a distance below or equal to *r* of any point of the point pattern, i.e. in our study, of tree Cartesian positions in a plot. The *L*(r) function makes it possible to distinguish three types of tree distribution patterns: regular (*L*(r)<0), random (*L*(r)≈0) or aggregated (*L*(r)>0). The statistical significance of the regular or aggregated pattern compared to a random pattern was determined by comparing the observed *L(r)* value to a 95% confidence envelope based on simulated *L*(r) values of a complete spatial randomness (CSR) of tree horizontal structure, simulated by 1,000 Monte Carlo simulations. We applied the spatial structure analysis separately to forest trees and fruit trees. Indeed, the *L(r)* function is applied to a set of individuals subjected to the same process of spatial distribution [33]. We assumed that this was the case for the two types of shade trees in the stands: forest trees and fruit trees. To mitigate edge effects (taken into account in our analyses by the isotropic edge correction as proposed in Ripley [32]), the outcome of the *L*(r) function should be interpreted for *r* values less than half or a quarter of the width of the plot [33]. Thus, *L*(r) was calculated for *r* values less than 12 m, i.e. less than 1/4 of 50 m. For statistical reasons, the *L*(r) function cannot be used on patterns at low stand densities [34]. Therefore, the *L*(r) function was calculated only in plots with at least 10 forest trees or 10 fruit trees per plot. The 12 values of *L*(r) calculated for forest trees (r from 1 m to 12 m) and the 12 values of *L*(r) calculated for fruit trees were used in two separate Principal Component Analyses (PCA). With respect with the Kaiser rule, we retained the PCA axes that explained at least 80% of the inertia [35]. Subsequently, the observation coordinates on these axes were used in two different hierarchical cluster analyses based on the Euclidean distance and Ward’s criterion [36]. This resulted in clusters of plots with a similar horizontal spatial structure (regular, random, or aggregated) for forest trees (*HSFo*) and for fruit trees (*HSFu*). Plots not included in the typologies due to low densities of forest or fruit trees were attributed to a modality denoted “Low density” in the *HSFo* and *HSFu* categorical variables.

### Pest and disease assessment

**Figure 2:**
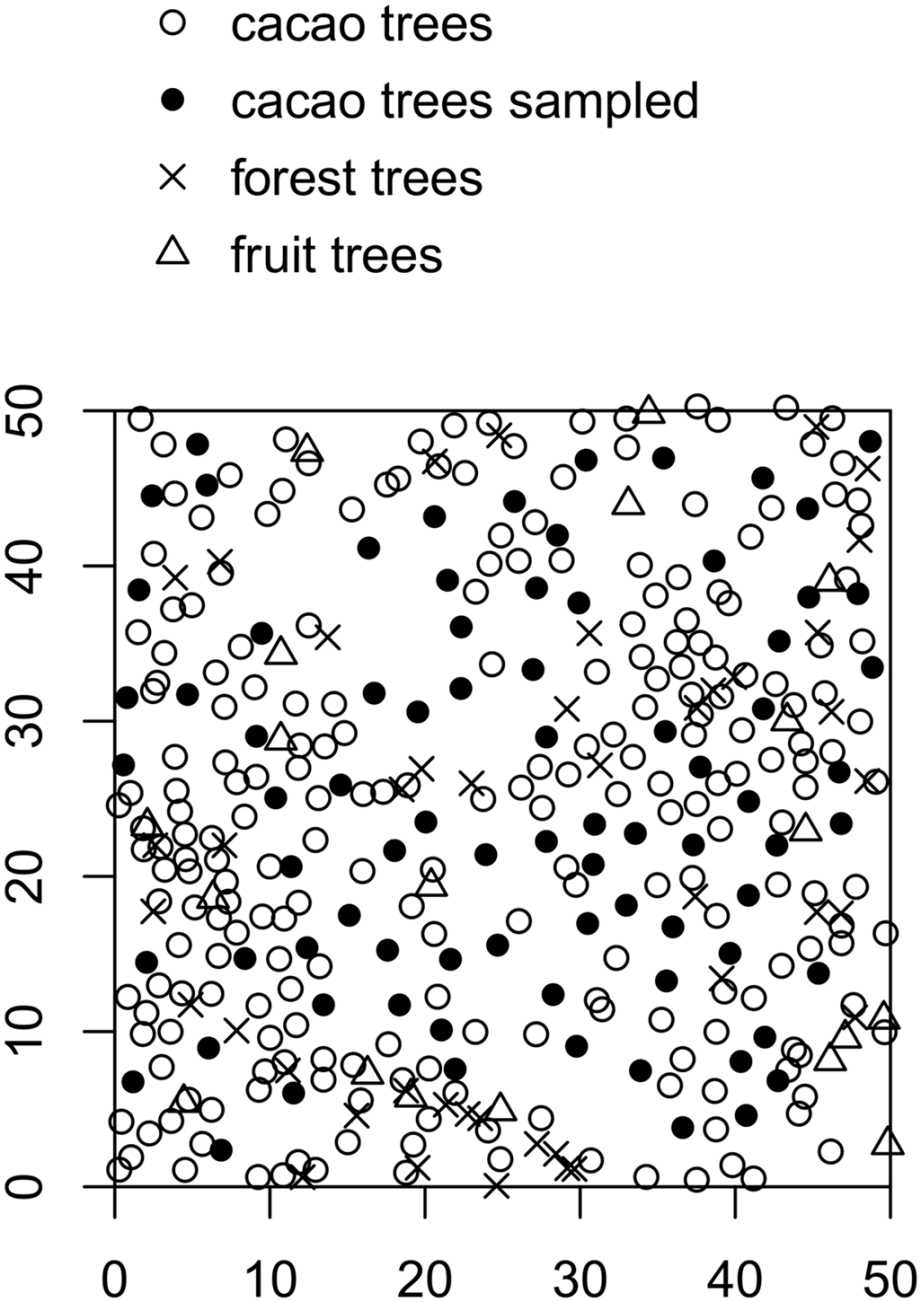
A sample map of a cacao agroforest plot studied.

In each plot, 80 adult cacao trees were selected for observations of the presence of sensitive tissues and for pest and disease monitoring. The trees were selected so as to be homogeneously distributed in the plot (Figure 2).

Plots were not treated with insecticide by farmers for the two years of the study. Mirid density was evaluated in September 2011 and August 2012, i.e. during the usual period of mirid outbreaks in the region [21]. For sampling, the 80 cacao trees were sprayed with an insecticide composed of 20 g/L of lambda cyhalothrin and 20 g/L of imidaclopride using a motorized mist blower (Stihl 420, Stihl AG, Waiblingen, Germany) equipped with a formulation pump and a 0.8 restrictor, and fitted with the baffle plate provided by Stihl adjusted to 25 mL/ha. Treatments were carried out at 6∶00 AM, when mirids are not yet very active to limit the risk of winged adults escaping. Seven hours after the treatment, dead insects were collected on 4×4 m plastic sheets positioned under the cacao trees before spraying. The collected insects were preserved in glass hemolysis tubes containing 70% alcohol. The French Agricultural Research Centre for International Development (CIRAD) and the Agricultural research institute for the development (IRAD) have granted permission. In the laboratory, the individual mirids belonging to *Sahlbergella singularis* collected under the 80 selected cacao trees in each plot were counted. For each year, mirid density per plot (*Dmir)* was estimated from the mean number of mirids per tree and the density of cacao trees per plot.

**Figure 3:**
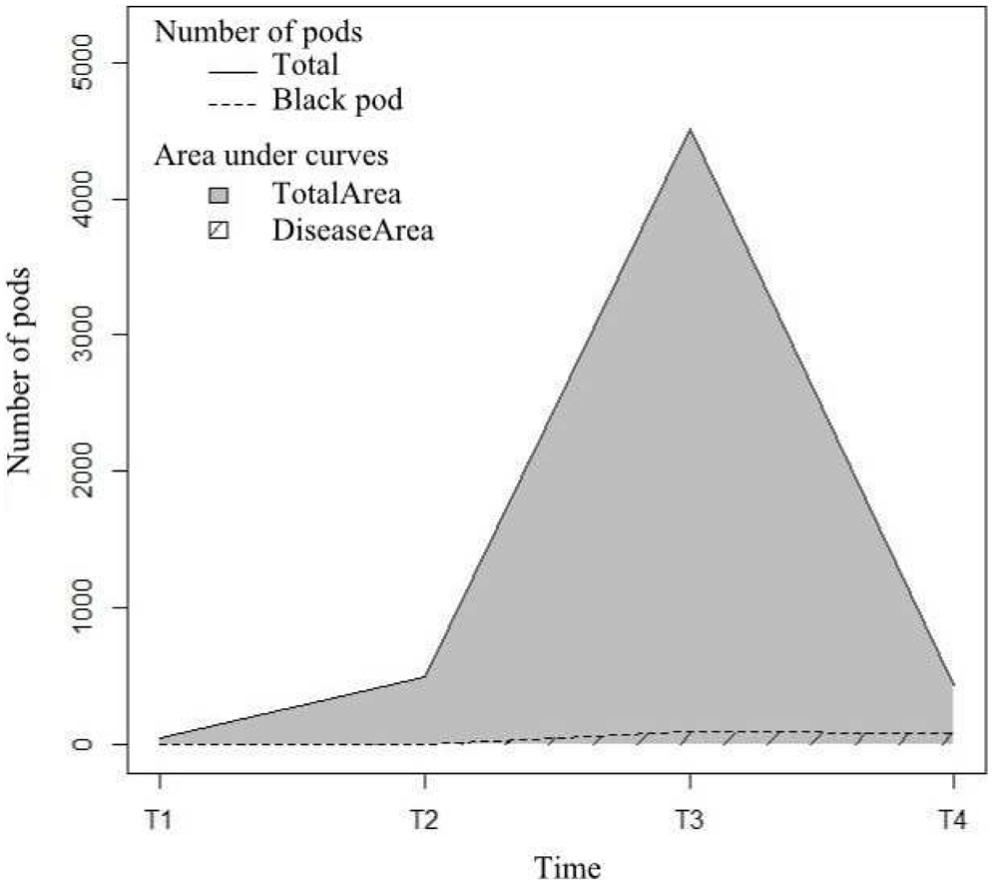
Number of total and damaged pods on the 80 cacao trees sampled in one plot. Number of pods from T1 (May 2012) to T4 (November 2012) on the 80 cacao trees sampled in the plot studied and mapped in Figure 2.

Data on disease damage were collected at four dates: T1: May 2012, T2: July–August 2012, T3: October 2012, T4: November 2012. At each date, healthy and diseased pods (infected by black pod) were counted on the 80 selected cacao trees. Only pods at least 10 cm in length were considered. For each plot, the mean of the total number of pods (healthy and diseased pods) and the mean of the number of diseased pods per tree were calculated for the 80 selected cacao trees and multiplied by the number of cacao trees present in the plot. Based on these values, for each plot, we plotted the curves of the total number of pods from T1 to T4 and the number of diseased pods over the same period (Figure 3). Next, black pod prevalence (*BPP*) was calculated using the formula: 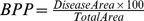, where *TotalArea* is the area under the total number of pods curve and *DiseaseArea* is the area under the number of diseased pods curve [18]. Fungicide treatments by farmers were homogeneous between plots with a recommended rate of one 50 g bag of Ridomil Gold plus 66 WP (Active ingredients: metalaxyl-M 6% and copper(1)oxide 60%) applied with a side lever knapsack sprayer containing 15 L water, four times between T1 to T4. Recorded differences between farmers’ practices did not have a significant effect on black pod prevalence (data not shown) and were therefore not taken into account in our analysis.

### Assessment of available sensitive tissue

We also describe pest and disease resources using variables that account for the amount of sensitive tissue available. Since black pod only attacks cacao tree pods and mirids preferentially attack the young shoots and pods, the attractiveness of a plot varied as a function of the amount of these sensitive tissues it contained. The amount of tissues sensitive to mirids was evaluated by the proportion of cacao trees with young shoots (called flushes) and the number of pods. For each plot, the flush presence variable (*Flush*) was the mean proportion of the 80 selected cacao trees with flushes during the study period, i.e. from T0 = September 2011 to T4 = November 2012. The number of pods available to mirids was estimated as the total number of pods, healthy and diseased, at the plot scale at T0 and T3 (the two periods when mirid outbreaks occur). The variable for pod production was denoted *Prod1* and is expressed as the number of pods.

The amount of tissues sensitive to *P. megakarya* was evaluated by the number of pods. For each plot, the number of pods was assessed using the value of the area under the curve of the total number of pods from T1 to T4 (Figure 3). The number of pods available for black pod was denoted *Prod2* and is expressed as fruit x day.

In all, 9 explanatory variables related to the host composition (*Abca*, *Alter*), shade tree spatial structure (*Dtot*, *Dsha, %Inter*, *HSFo*, *HSFu*), sensitive tissue amount (*Flush*, *Prod1* or *Prod2*) were calculated to explain mirid density (*Dmir*) or black pod prevalence (*BPP*) at the plot scale (see Table 1 for the list of variables).

**Table 1:** List of variables used to describe the 20 cacao agroforests.

Categories	Variables	Code	Unit or modalities	Min	Max	Mean	Transf.
Mirid pest	Mirid density2011/12	*Dmir*	number of ind./ha	0	842	117	log
Black poddisease	Black podprevalence	*BPP*	%	0	5	1	sqrt
Host composition“resourceHypothesis”	Cacao abundance	*Abca*	%	74	94	84	-
	Alternative host	*Alter*	Presence				
			Absence				
Sensitive tissue“resourceHypothesis”	Number of pods2011/12	*Prod1*	number of pod/ha	78	22178	8497	sqrt
	Number of pods2012	*Prod2*	number of pod x day	80.10^4^	31.10^5^	21.10^5^	
	Flush presence	*Flush*	-	0.2	0.5	0.3	sqrt
Spatial structure“microclimateHypothesis”	Total plant density	*Dtot*	number of trees/ha	580	1660	1129	-
	Density ofassociatedshade trees	*Dsha*	number of shade trees/ha	40	168	97	log
	% intermediate trees	*%Inter*	%	18	92	51	sqrt
	Spatial structure offorest trees	*HSFo*	Low density				
			Aggregated				
			Random				
	Spatial structure offruit trees	*HSFu*	Low density				
			Random				
			Regular				

Variables for density and number of pods are presented at the hectare scale but are used at the plot scale (1/4 ha) in statistical analyses.

### Statistical models

Statistical models were developed to explain Ys variables, i.e. mirid density and black pod prevalence, by explanatory variables Xs for host composition, sensitive tissue availability and tree spatial structure. The approach we used comprised three main steps:

1. Ys and Xs variables were log or square-root transformed to respect the normal distribution assumption (Table 1). Variation in mirid density between 2011 and 2012 was compared by ANOVA. No significant effect was found (*F* = 2.10, *P* = 0.16). Consequently, the data for 2011 and 2012 were pooled for subsequent analysis.
2. Correlations between the Xs explanatory variables were tested using the Spearman rank correlation test for correlations between continuous variables and the Kruskal-Wallis test with the Dunn correction for pairwise comparisons for correlations between continuous and categorical variables. Some explanatory variables were not independent, with Spearman’s correlation coefficient values ranging from −0.54 to 0.50. Therefore, a hierarchical partitioning protocol [37] was used to examine the independent effect of each explanatory variable on mirid and black pod variables. Two hierarchical partitioning protocols were used: (i) one to explain mirid density (*Dmir*) with the variables *Abca*, *Alter*, *Prod1*, *Flush*, *Dtot*, *Dsha*, *%Inter*, *HSFo* and *HSFu* and (ii) one to explain black pod prevalence (*BPP*) with the variables *Abca*, *Prod2*, *Dtot*, *Dsha*, *%Inter*, *HSFo* and *HSFu*. Hierarchical partitioning is a method that considers all possible models in a multiple regression setting to identify the most likely causal factors (explanatory approach). The independent effect (denoted I_i_) of an explanatory variable X_i_ on Y is then obtained by averaging all the increases in fit generated by including the variable X_i_ in all the models in which X_i_ appears. R-squared, which expresses the Y variance explained by a model, was used as goodness-of-fit measure. The joint effect J_i_ of the X_i_ variable (effect caused jointly with the other Xs variables) is obtained by subtracting I_i_ from R_i_, with R_i_ the R-squared of the model explaining Y with only X_i_. For each X_i_ variable, the independent and joint contributions are expressed as a percentage of the total explained variance R_i_, with R_i_ = I_i_+J_i_. The significance of independent effects is evaluated by Mac Nally’s Z-scores using a randomization test with 1,000 iterations [38]. There is no measurement of the significance of joint effects. However, it is important to not only look at the independent “explanatory” power (I) of variables but also the ratio between I and J. Because, even if X_i_ has an apparently high independent effect on Y (great I_i_), if X_i_ appeared to have complex inter-relationships with other explanatory variables (great J_i_), then manipulating X_i_ alone may not have the desired outcomes [37].
3. Hierarchical partitioning does not provide information on the direction of the relationship (positive or negative) between the Y variable and each explanatory variable. Therefore, to test the effect of microclimatic hypothesis variables on pest density or disease prevalence after controlling for the effect of resource hypothesis variables: (i) we built all the possible linear models that explain Y with the combination of variables of the resource hypothesis, i.e. the variables describing host composition and the sensitive tissue availability, (ii) the best model, with the smallest AIC (Akaike Information Criterion [39]) was chosen, (iii) we used the residuals of this model and we tested their relationship (positive or negative) with each variable of the microclimatic hypothesis, i.e. *Dtot*, *Dsha*, *%Inter*, *HSFo* and *HSFu*. To analyze the relationship, a Pearson correlation test (for continuous explanatory variables) or an ANOVA (for categorical explanatory variables) was performed. The same procedure was applied to study the effect of resource hypothesis variables after controlling for the effect of the microclimatic hypothesis [40]–[41].

Statistical analyses were performed with the packages “stats” for statistical hypothesis testing (e.g. ANOVA, Spearman rank correlation test, Kruskal-Wallis test), “ads” for the point pattern analyses (*L(r)* function), “FactoMineR” for the Principal Component Analyses and “hier.part” for the Hierarchical Partitioning protocol of the R software 2.15.0 [42]–[43].

## Results

### Vegetation composition and spatial structure

The relative abundance of cacao tree (*Abca*) ranged from 74% to 94% with a mean of 84% with respect to all plants 2 m-tall or over. In 11 plots, at least one of the alternative mirid hosts (*Alter*), i.e. *Ceiba pentandra*, *Cola acuminata*, *Cola lateritia* or *Sterculia rhinopetala,* was present (Table 1).

The total density of plants 2 m-tall or over (*Dtot*) ranged from 580 to 1,660 with a mean of 1,129 individuals per hectare (Table 1). Shade tree density (*Dsha*), i.e. only associated trees that were taller than the cacao trees, ranged from 40 to 168 with a mean of 97 shade trees per hectare (Table 1). The percentage of shade trees belonging to the intermediate stratum (*%Inter*) ranged from 18 to 92 with a mean of 51%. Concerning the horizontal structure of the forest trees (*HSFo*), five plots had a forest tree density <10 individuals per plot (40 trees per hectare, Table 2) and were classified as “low density”. In the 15 other plots, forest tree density ranged from 48 to 156 trees per hectare. Seven of these plots displayed a significant aggregated structure of forest trees, with positive values of the *L(r)* function > the 95% confidence envelope for at least one *r* value (Table 2). In the eight other plots, forest trees displayed a structure that did not significantly differ from a random distribution. Concerning horizontal structure of the fruit trees (*HSFu*), eight plots had a fruit tree density of less than 10 individuals per plot (40 trees per hectare) and were classified as “low density”. In the 12 other plots, fruit tree density ranged from 40 to 104 trees per hectare. Three plots had significant negative values of the *L(r)* function, and a trend towards a regular distribution was observed in four plots. In the five remaining plots, fruit trees displayed a structure that did not significantly differ from a random distribution.

**Table 2:** Information on plots concerned by the variable forest tree horizontal structure (*HSFo*).

*HSFo* modalities	Low density	Aggregated	Random
Number of plots	5	7	8
Density of forest trees	<10 ind./plot	>10 ind./plot	>10 ind./plot
*L(r)* curves *outsideconfidence envelope	No calculation of *L(r)* dueto low forest treedensities	*L(r)*>0	*L(r)* = 0
Log(*Dmir*) ANOVA:F = 3.9* Tukey HSD^1^	3.5 a	2.7 ab	2.1 b

1 means with different letters are significantly different, P<0.05.

### Factors affecting mirid density

**Figure 4:**
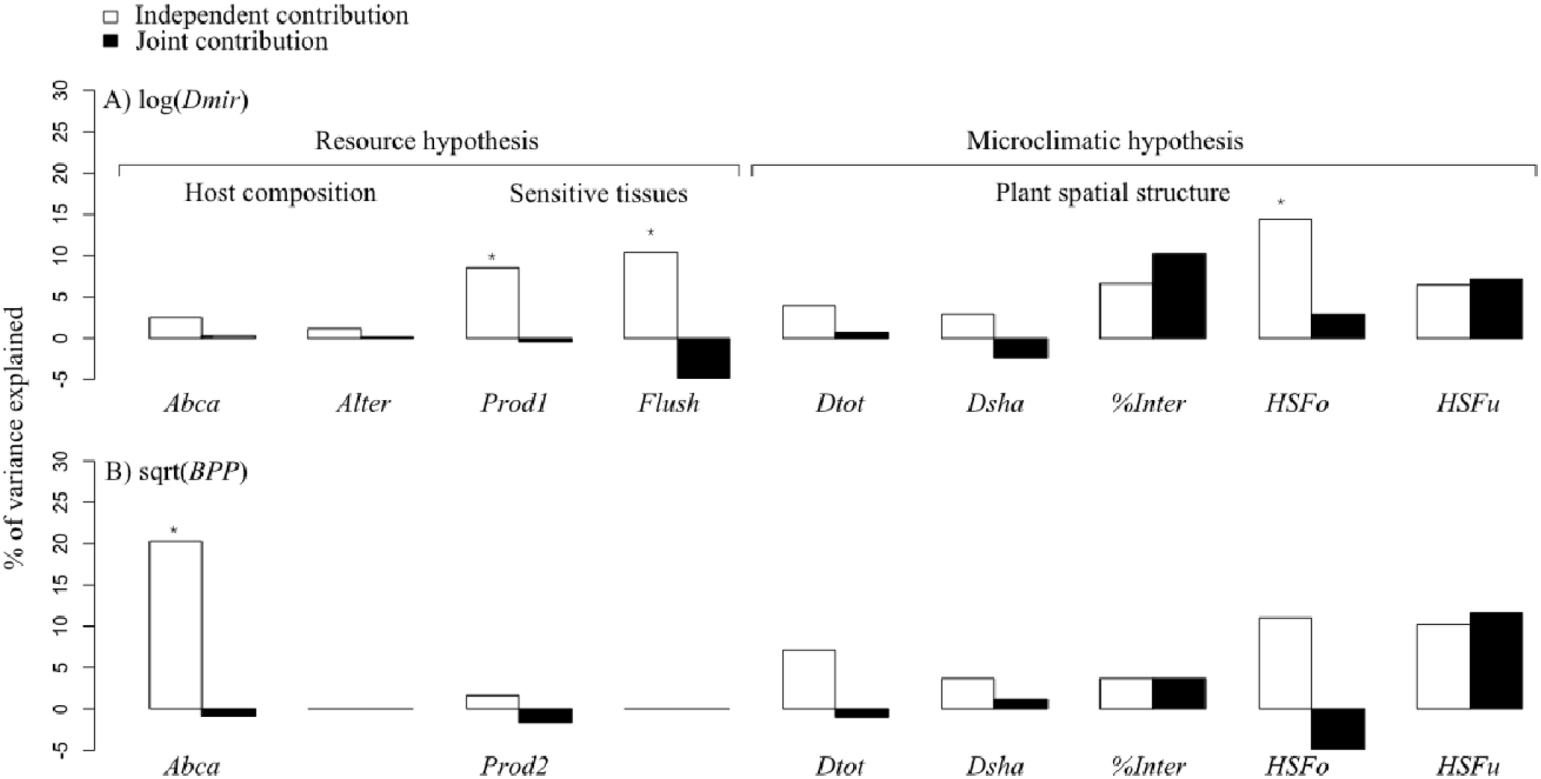
Independent and joint contribution (% of variance explained) of explanatory variables on mirid density and black pod prevalence. Results of the hierarchical partitioning analyses of A) mirid density (*Dmir*) and B) black pod prevalence (*BPP*). Significant independent contributions of explanatory variables are indicated by *(Z-score value>1.65, determined by randomization tests with 1,000 iterations). See Table 1 for the definition of the variables.

Mirid density ranged from 0 to 842 *Sahlbergella singularis* individuals per hectare with a mean of 117 mirids per hectare (Table 1). The variables sensitive tissue availability and shade tree spatial structure partially explained mirid density at the plot scale (Figure 4A).

The presence of flushes (*Flush*) and the number of pods (*Prod1*) had significant independent effects on mirid density (*Dmir*). They explained respectively 10.4% and 8.5% of mirid density variance independently of the other variables (Figure 4A). Flush presence and the number of pods were significantly positively correlated with mirid density (Table 3), after controlling for the effects of variables for tree spatial structure.

**Table 3:** Table of correlations.

Categories	Codes	Modalities	*Dmir*	*BPP*
Host composition	*Abca*		−0.02	−0.36
	*Alter*		0.00	-
		Presence	0.04	-
		Absence	−0.05	-
Sensitive tissues	*Flush*		0.25	-
	*Prod1*		0.34	-
	*Prod2*		-	−0.01
Spatial structure	*Dtot*		0.39	0.39
	*Dsha*		0.10	−0.02
	*%Inter*		0.33	0.20
	*HSFo*		0.13	0.21
		Low density	0.64	0.35
		Aggregated	0.03	−0.27
		Random	−0.43	0.02
	*HSFu*		0.11	0.17
		Low density	−0.44	−0.24
		Random	0.02	0.04
		Regular	0.48	0.24

Pearson coefficients and R^2^ ANOVA values respectively, between continuous or categorical explanatory variables and residuals of models for mirid density (*Dmir*) or black pod prevalence (*BPP*) after controlling for the effects of the other hypotheses. The mean of residual values are indicated for modalities of categorical variables. See Table 1 for code and definition of variables.

The horizontal structure of forest trees (*HSFo*) had a significant independent effect on mirid density (*Dmir*) and explained 14.5% of mirid density variance independently of the other variables (Figure 4A). Mean mirid density at the plot scale decreased when *HSFo* went from “Low density” to “Aggregated”, and then from “Aggregated” to “Random” structure, after controlling for the effects of host composition and the availability of sensitive tissue (Table 2 and Table 3).

None of the host composition variables had a significant independent effect on mirid density (Figure 4A).

The joint effects of *Flush, Prod1* and *HSFo* with the other explanatory variables on mirid density were lower than their independent effects (Figure 4A).

### Factors affecting black pod prevalence

Black pod prevalence (*BPP*) ranged from 0 to 5% infected pods of the total number of pods in 2012 at the plot scale with a mean of 1% (Table 1).

Among the host composition variables, cacao tree abundance (*Abca*) had a significant independent effect on black pod prevalence and explained 20.3% of the variance independently of the other variables (Figure 4B). Cacao tree abundance was negatively correlated with black pod prevalence (Table 3), after controlling for the effects of variables for tree spatial structure. The joint effect of *Abca* and the other explanatory variables on black pod prevalence was lower than its independent effect (Figure 4).

Neither sensitive tissue availability, i.e. the number of pods, nor the variables related to tree spatial structure had a significant independent effect on black pod prevalence (Figure 4B).

## Discussion

The aim of this study was to assess the independent effects and the relative importance of different variables related to resource availability and microclimatic variation that explain pest and disease occurrence in real complex agro-ecosystems, at the plot scale.

Our study showed that some characteristics of vegetation structure related to the different study hypotheses influence pest and disease occurrence in the farmers’ agroforests studied here.

### Concerning mirids

Our hypothesis was that tree spatial structure influences mirid density through microclimatic variations. We assumed that the low density of shade trees or the microclimatic heterogeneity generated by the aggregation of shade trees could increase mirid density at the plot scale due to the higher density of mirids on cacao trees in direct sunlight than on cacao trees in the shade [21]. We also expected that when there were relatively more shade trees in the top stratum than in the intermediate stratum, mirid density would be reduced due to the decrease in mean transmitted light [21–12]. Concerning plant spatial structure variables, our results showed that only the variable horizontal structure of forest trees impacted mirid density independently of other variables. Mirid density decreased when a minimum number of randomly distributed forest trees were present compared with an aggregated distribution or when the density of forest trees was low. Babin et al. [21] found that mirid pockets, which are areas highly infested by mirids, were generally located in the sunniest zones of plots. In cacao agroforests, large forest trees, unlike fruit trees, tend to homogenize the distribution of the light resource and consequently limit the development of mirid pockets [21]. Our results confirm that the presence of forest shade trees is correlated with a decrease in mirid density. Several architectural characteristics distinguish forest trees from fruit trees. The most obvious difference is their height as only forest trees reached the top canopy level. However, this difference is probably not the only one involved, since otherwise the variable vertical structure (*%Inter*) would have been significant rather than the variable forest tree horizontal structure (*HSFo*). Thus, the difference in architecture of forest trees which generally have a larger and more porous canopy than fruit trees (a trend revealed by our data, data not shown) could thus also explain why forest trees are most likely to provide more uniform shading. Moreover, the reduction in mirid density is not only observed when forest trees are left standing in cacao agroforests, but this reduction appears to be more effective when forest trees are randomly distributed rather than aggregated. The interaction between the spatial organization of individuals and the distribution of the light resource has only rarely been studied in complex plant ecosystems. Using simulations, Martens et al. [12] showed that the variance in understory light is dependent on the spatial patterns of trees, with an increase in the variance of understory light when the horizontal structure of canopy trees progresses from random to aggregate. Thus, forest tree aggregation is likely to introduce heterogeneity in light distribution, with an alternation of shaded (under aggregates) and sunny (outside aggregates) environments, with sunny environments favoring the development of mirid pockets. Regular patterns of forest trees were not observed at our study site whereas they were observed for fruit trees. This is probably due to differences in the management of forest trees, which are left over from the previous forest ecosystem, compared with fruit trees, which are mainly planted by farmers [19]–[44]. In a previous study, we showed that compared with other horizontal structures or low density, regular forest tree structure reduced frosty pod rot (caused by *Moniliophthora roreri*) prevalence on cacao pods in an agroforest in the Talamanca region in Costa Rica [18]. The results we obtained concerning mirid density and the above result concerning frosty pod rot prevalence underline the potential of promoting forest tree horizontal structure for the agricultural management of cacao pests and diseases. It is interesting to note that in our study, the classical shade tree density or total tree density variables had no significant effect on pest density, whereas the variable tree horizontal structure, which is rarely taken into account, did. Our results thus open new perspectives for agro-ecological research on the regulation of pest and disease by shading through the tree spatial structure.

We also assumed that host composition and sensitive tissue availability influence mirid density through variations in resources. Agroforests are often described as complex agro-ecosystem with significant planned and spontaneous plant diversity [4]–[19]. We studied real agroforests and one of our observations was that cacao agroforests in the Lékié department varied little with respect to the abundance of cacao trees: cacao tree relative abundance ranged from 74% to 94%. This range may not be large enough to empirically test the resource dilution mechanism with the host abundance as proxy to describe the resource amount. Plus, the amount of available sensitive tissue is rarely taken into account in studies on the dilution mechanism. However, our study showed that the amount of sensitive tissue is probably a more valid proxy for describing the resources available for pests and diseases than host composition. Indeed, our results showed that, unlike cacao tree abundance, pod and flush production both had significant independent effects on mirid density. This effect is in agreement with the dilution mechanism since a decrease in the resource (here the amount of sensitive tissue) leads to a decrease in the pest density. In a previous study, we showed that the amount of sensitive tissue was more important than host composition in explaining variations in cacao tree disease [18]. Therefore, besides host abundance, it is important to include the availability of sensitive tissues to identify the effect of variations in resource availability on the pest and disease in real multispecies agro-ecosystems. This variable is particularly important since it makes it possible to determine whether microclimatic variation has a direct or indirect effect on the pest and disease, through variations in plant growth.

A major aim of this study was also to disentangle the effects of the mechanisms involved in the “microclimatic” and the “resource” hypotheses on mirid density in cacao agroforests. Our results showed that the variables forest tree spatial structure and the amount of sensitive tissue both had independent effects on mirid density, thus indicating that hypotheses concerning both the “microclimatic” and “resource” mechanisms may be involved in mirid infestation. Crucial life history traits of cocoa mirids could enlighten us: they are photophobic insects [23], yet at the plot level, they are often located in the sunniest environments [21]. To explain this contradiction, Babin et al. [21] suggested that higher resource availability related to flush intensity, which is greater in cacao trees exposed to sunlight, may attract and allow the development of larger mirid populations. From this assumption, we could conclude that, for cocoa mirids, “microclimatic” mechanisms may act indirectly through “resource” mechanisms. But our results also showed that the effects of the structure of shade trees and the amount of available resources were independent. Moreover, there was no significant difference in the proportion of cacao trees with flushes and in the number of pods between plots with different forest tree horizontal structure (unpublished data). Our study thus did not reveal a relationship between “resource” and “microclimatic” mechanisms. Consequently it is difficult to validate or invalidate the hypothesis that mirids are present in the sunniest areas because of the larger amount of sensitive tissues. A further study that considers the different variables at the cacao tree scale, (and not only at a plot scale as was the case in our study), would help answer this question. However, in terms of tradeoff between pest regulation and pod production services, our results are particularly interesting because we showed that mirid density could be reduced by optimizing the design of tree associations without affecting average pod production at the plot scale.

### Concerning black pod

The low percentages of black pod prevalence (*BPP*) in our study plots, which did not reach more than 5% of the production, partly due to homogenized treatment with fungicides, preclude any definitive conclusions. Indeed, without treatment, yield losses as high as 50% are commonly observed and when conditions are favorable, yield losses can reach 80% [45]–[46]. We also presume that the year our observations were conducted was a year with relatively low losses and also that the four point observations of prevalence in the year may also underestimate real losses accumulated over entire year. Nevertheless, despite the fungicide treatment in the plots and the low prevalence rates observed, the host composition effect is probably a particularly strong effect. For that reason, we discuss this effect below, as we believe that it may be even more pronounced in cacao plots without fungicide treatments.

One of our hypotheses was that host composition and sensitive tissue availability influences black pod prevalence through resource variation. In our study plots, black pod prevalence was only explained by cacao tree abundance, independently of other variables, and black pod prevalence increased with a decrease in cacao tree abundance. Based on the “resource dilution” mechanism the opposite result would be expected, in other words a decrease in host tree abundance should have led to a decrease in disease prevalence [47]. Other hypotheses could be proposed to explain this result. Firstly, the relation that exists between the age of cacao agroforests and vegetation structure linked to the farmer’s choice of management: On the one hand, old cacao trees that die are not systematically replaced with other cacao trees, or if they are replaced, the fact the cacao tree seedlings were small meant they were not taken into account in our mapping (which only included plants over 2 m in height). Moreover, cacao tree seedlings are sometimes planted together with Musaceae to provide shade during seedling growth [48] and also to provide an income for farmers from the sale of Musaceae fruits. This type of management can lead to a decrease in cacao tree density but also in cacao relative abundance linked to the age of the plantation. On the other hand, an increase in the age of the cacao plot may also lead to an increase in the amount of primary inoculum of *P. megakarya* in the plot, which has accumulated over the years. The negative relationship between cacao tree abundance and black pod prevalence could thus be partly explained by these two effects of the age of the plantation. Secondly, we could also expect that the increase in cacao tree abundance, which was negatively correlated with plant species richness in our plots (unpublished data), would lead to a decrease in fauna associated with non-cacao trees, like ants, at the plot scale. For example, ants of the genus *Crematogaster* are very common in cacao agroforests in our study region, and move in large numbers on cacao trees, where they tend honeydew hemiptera and prey on various arthropods [49]. These ants nest in dead wood or in hollow branches and trunks, so that they benefit from the presence of big forest trees [50]. These ants, like other insects, are assumed to play a determining role in the propagation of black pod by transporting soil particles that contain *Phytophthora* spores [51]–[52]. The potential decrease in the population of ants nesting in big shade trees, due to the increase in the relative abundance of cacao trees, could thus explain our results.

Another of our hypotheses was that the spatial structure of shade trees influences black pod prevalence by causing microclimatic variations. Concerning black pod prevalence, we assumed that the low density of self shading (*Dtot*) and of shade trees (*Dsha*) could reduce black pod prevalence at the plot scale due to aeration of the plot and a concomitant decrease in relative humidity [22]. We also expected that a decrease in the percentage of shade trees in the intermediate stratum (rather than in the top stratum) would decrease black pod prevalence due to more even rain interception and better plot aeration in the cacao tree stratum [22]. However, our results showed that, in our study, none of the variables related to plant spatial structure had an independent effect on disease prevalence. Mfegue [53] found no relationship between shade classes and black pod incidence either. In a previous study in Costa Rican cacao agroforests, we showed that only a regular spatial structure of forest trees reduced frosty pod rot prevalence (caused by *Moniliophthora roreri*) on cacao pods in comparison with plots with a low density of forest trees [18]. Like *M. roreri*, *P. megakarya* is primarily a cacao pod pathogen and the germination of these two pathogens is favored by high humidity, and hence by the presence of shade trees [52]. However, they have two different main modes of dispersal, *M. roreri* by wind and *P. megakarya* by rain. Consequently, shade reduces the spore dispersal of *M. roreri* while its impact on the spore dispersal of *P. megakarya* is not yet known. To control the development of frosty pod rot, it is generally advised to provide moderate and uniform shade at the plot scale, while to control black pod development, it is generally advised to reduce shade. In the Costa Rican study, in the case of *M. roreri,* we showed that although lower disease prevalence was not automatically achieved by conserving forest trees, this reduction was effective only if forest trees were regularly distributed throughout the plot. In the plots in the study area in Cameroon, the lack of impact of the variable tree spatial structure on black pod prevalence is certainly related to the generally high density of shade trees, more dense than the shade tree pattern that could reduce the development of the disease (Table S1).

This study also aimed to disentangle the effects of the mechanisms involved in the “microclimatic” and the “resource” hypotheses on the prevalence of black pod disease in cacao agroforests. One possible explanation for the surprising negative relationship between cacao tree abundance and black pod prevalence could be an increase in the relative abundance of shade trees due to the decrease in the abundance of the cacao trees. This increase in the relative abundance of shade trees could create a shadier environment, which supposedly increases black pod development. However, this explanation can be excluded, since if this were the case, one of the tree spatial structure variables, such as shade tree density or total tree density, would be significant in the hierarchical partitioning analysis. Due to the very low joint effect of the variable cacao tree abundance in the hierarchical partitioning analysis, to find an explanation for this result, we need to look for a variable that was not taken into account in our analysis and is correlated with cacao tree abundance, as proposed in the previous paragraph with regard to the age of the plot and the effect of inoculum accumulation.

Further studies, including studies involving classical variables known to influence the disease (e.g. inoculum potential) and plant structure variables, are needed to elucidate the natural mechanisms involved in the regulation of black pod. However, our results already show that optimizing host composition could be an effective way to decrease black pod prevalence.

### Joint agro-ecological management implications for mirids and black pod disease and future outlook

One of the recommendations for the control of mirids is to maintain a uniform shade level in cacao tree plantations, while one of the recommendations for the control of black pod is to avoid excessive shade [21]–[22]. “Thus, to be effective, shade management strategies have to find a balance between shade conditions unfavourable for both mirids and black pod [21]”. The present study provides new information on how tree structure characteristics affect the development of the pest and the disease. This is important, since knowledge of such characteristics will help improve pest and disease management strategies. Forest trees are expected to be better shade trees to reduce the occurrence of both mirids and black pod [21]–[54]. In our study, we confirmed that forest trees are better than fruit trees because they appear to provide better shade conditions for mirid control. What is more, our results suggest that reduced pest density is not automatically achieved by preserving forest trees. Indeed, we observed that aggregated forest trees are less efficient than a more randomised tree pattern probably because the latter homogenizes the light under the canopy. Our results also showed that variation in the spatial structure of forest trees did not significantly affect black pod prevalence at the plot scale. Consequently, in our study area, it appears that an agro-ecological approach to mirid and black pod management could be the preservation of natural resources, in this case native forest trees, but under farmer management. However, the preservation of forest trees in cacao agroforests could also favor the presence of a certain fauna, for example ants, that may be implicated in the dissemination of black pod. Thus a more detailed understanding is needed of the causes of the relationship between black pod prevalence and cacao tree abundance, along with studies that involve classical variables known to influence the disease and plant structure variables, are important.

Based on previous observations, we might expect similar results with respect to the impact of the spatial structure of canopy trees on biodiversity in general [55] and on other pests and diseases in particular. Indeed, we also assume that the importance of the spatial structure of shade trees for mirid or frosty pod rot [18] regulation also applies in coffee plantations, where leaf rust caused by *Hemileia vastatrix* is less severe under moderate shade [8]. However, the horizontal spatial structure of shade trees is a structural characteristic that has not received adequate attention to date, and the description of tree patterns and their impact on ecosystem functioning, particularly on pest and disease regulation services, need to be studied in more detail.

Many studies on the relationships between plant diversity and pest or disease occurrence have focused on the resource dilution mechanism. However, the main resource for the pests and diseases is often also the main financial resource for farmers, as is the case of the cacao tree and its fruits here. This study, which takes into account microclimatic and resource mechanisms, showed that the effect of the spatial structure of forest trees is independent of the amount of sensitive tissue (leaves and pods) and therefore provides interesting information on the tradeoff between pest regulation and pod production services.

## Supporting Information

Table S1 **Values of each variable for the 20 plots studied.**
(DOCX)
