# Supplementary material for: Tree Spatial Structure, Host Composition and Resource Availability Influence Mirid Density or Black Pod Prevalence in Cacao Agroforests in Cameroon
Source: PLoS One. 2014 Oct 14;9(10):e109405. doi: 10.1371/journal.pone.0109405 (PMC4196851; doi:10.1371/journal.pone.0109405)
Supplement: Table S1 — Values of each variable for the 20 plots studied. (DOCX) [file pone.0109405.s001.docx]

| Plot | *Dmir*  2011 | *Dmir*  2012 | *BPI* | *Abca* | *Alter* | *Flush* | *Prod1* 2011 | *Prod1* 2012 | *Prod2* | *Dtot*  (ha) | *Dsha* (ha) | *%Inter* | *HSFo* | *HSFu* |
| --- | --- | --- | --- | --- | --- | --- | --- | --- | --- | --- | --- | --- | --- | --- |
| 1 | 10.6 | 0.0 | 1.7 | 79.2 | Present | 0.27 | 3,435.3 | 7236.9 | 11.10^5^ | 888 | 80 | *40* | Random | Regular |
| 2 | 48.4 | 69.8 | 0.3 | 87.0 | Present | 0.37 | 3,132.6 | 10185.9 | 15.10^5^ | 1000 | 88 | 45.5 | Random | Regular |
| 3 | 63.0 | 109.8 | 0.6 | 85.9 | Present | 0.37 | 3,368.7 | 14051.6 | 21.10^5^ | 760 | 80 | 35.0 | Aggregated | Low density |
| 4 | 0.0 | 34.3 | 0.0 | 89.9 | Absent | 0.32 | 8,475 | 18213.9 | 25.10^5^ | 1472 | 112 | 39.3 | Random | Random |
| 5 | 57.4 | 22.4 | 0.2 | 88.6 | Absent | 0.53 | 2,125.5 | 5853.3 | 80.10^4^ | 952 | 72 | 33.3 | Random | Low density |
| 6 | 0.0 | 0.0 | 0.3 | 93.5 | Present | 0.27 | 5,771.7 | 8260.1 | 16.10^5^ | 1328 | 56 | 28.6 | Random | Low density |
| 7 | 13.7 | 54.2 | 1.2 | 82.9 | Present | 0.21 | 78.3 | 15554.3 | 21.10^5^ | 1200 | 156 | 17.9 | Aggregated | Low density |
| 8 | 21.0 | 83.1 | 0.1 | 90.6 | Absent | 0.51 | 1,059.75 | 16640.0 | 25.10^5^ | 580 | 40 | 30.0 | Low density | Low density |
| 9 | 15.6 | 152.0 | 1.9 | 73.9 | Present | 0.28 | 109.2 | 12341.3 | 16.10^5^ | 756 | 124 | 64.5 | Aggregated | Random |
| 10 | 48.3 | 49.5 | 0.3 | 86.6 | Present | 0.26 | 11,028.5 | 12124.4 | 26.10^5^ | 1392 | 76 | 26.3 | Aggregated | Low density |
| 11 | 48.8 | 0.0 | 0.0 | 80.7 | Absent | 0.29 | 1,175 | 10992.2 | 15.10^5^ | 896 | 128 | 53.1 | Aggregated | Low density |
| 12 | 33.4 | 74.9 | 2.1 | 79.6 | Present | 0.16 | 1,971 | 22177.6 | 29.10^5^ | 1320 | 136 | 67.6 | Random | Random |
| 13 | 72.1 | 281.0 | 0.1 | 83.6 | Absent | 0.29 | 1,657.9 | 21967.8 | 31.10^5^ | 1236 | 80 | 80.0 | Low density | Regular |
| 14 | 21.4 | 325.3 | 2.8 | 81.7 | Absent | 0.47 | 463.5 | 12614.3 | 19.10^5^ | 912 | 64 | 87.5 | Low density | Regular |
| 15 | 40.0 | 224.0 | 0.5 | 84.7 | Absent | 0.43 | 949 | 16722.1 | 25.10^5^ | 1168 | 112 | 67.9 | Random | Random |
| 16 | 67.1 | 78.3 | 0.1 | 78.1 | Present | 0.33 | 1,831.95 | 17796.5 | 26.10^5^ | 908 | 72 | 55.6 | Aggregated | Low density |
| 17 | 94.9 | 842.4 | 0.6 | 92.9 | Absent | 0.37 | 912.6 | 21326.6 | 30.10^5^ | 1488 | 60 | 92.3 | Low density | Regular |
| 18 | 331.2 | 229.8 | 4.9 | 82.7 | Present | 0.42 | 3,370.4 | 18953.0 | 26.10^5^ | 1660 | 88 | 68.2 | Low density | Regular |
| 19 | 213.3 | 259.2 | 0.7 | 79.8 | Absent | 0.29 | 665.6 | 6763.5 | 93.10^4^ | 1176 | 140 | 68.6 | Aggregated | Regular |
| 20 | 146.3 | 439.6 | 2.2 | 80.5 | Present | 0.33 | 427.95 | 18077.1 | 23.10^5^ | 1484 | 168 | 26.2 | Random | Random |

**Table S1: Values of each variable for the twenty plots studied.**
